# Antifungal Effect of Poly(methyl methacrylate) with Farnesol and Undecylenic Acid against *Candida albicans* Biofilm Formation

**DOI:** 10.3390/ma17163936

**Published:** 2024-08-08

**Authors:** Milica Išljamović, Debora Bonvin, Milena Milojević, Simona Stojanović, Milan Spasić, Branislava Stojković, Predrag Janošević, Suzana Otašević, Marijana Mionić Ebersold

**Affiliations:** 1 Powder Technology Laboratory, Institute of Materials, Ecole Polytechnique Fédérale de Lausanne, 1015 Lausanne, Switzerland; 2 Department of Dental Health Care, Health Center Niš, 18000 Niš, Serbia; 3 Department of Pharmacy, Faculty of Medicine, University of Niš, Blvd. Dr Zoran Djindjić 81, 18000 Niš, Serbia; 4 Department of Oral Surgery, Faculty of Medicine, University in Niš, Blvd. Dr Zoran Djindjić 81, 18000 Niš, Serbia; 5 The Niš Dental University Clinic, Blvd. Dr Zoran Djindjić 52, 18000 Niš, Serbia; 6 Department of Preventive and Pediatric Dentistry, Faculty of Medicine, University of Niš, Blvd. Dr Zoran Djindjić 81, 18000 Niš, Serbia; 7 Department of Orthodontics, Faculty of Medicine, University of Niš, Blvd. Dr Zoran Djindjić 81, 18000 Niš, Serbia; 8 Department of Microbiology and Immunology, Faculty of Medicine, University of Niš, Blv. Dr Zoran Djindjić 81, 18000 Niš, Serbia; 9 Public Health Institute Niš, Blvd. Dr Zoran Djindjić 50, 18000 Niš, Serbia

**Keywords:** undecylenic acid, farnesol, PMMA, denture, *C. albicans*, filamentation, biofilm, antimicrobial surface, *Candida*-associated denture stomatitis

## Abstract

The control of *Candida albicans* biofilm formation on dentures made of poly(methyl methacrylate) (PMMA) is an important challenge due to the high resistance to antifungal drugs. Interestingly, the natural compounds undecylenic acid (UDA) and farnesol (FAR) both prevent *C. albicans* biofilm formation and could have a synergetic effect. We therefore modified PMMA with a combination of UDA and FAR (UDA+FAR), aiming to obtain the antifungal PMMA_UDA+FAR composites. Equal concentrations of FAR and UDA were added to PMMA to reach 3%, 6%, and 9% in total of both compounds in composites. The physico-chemical properties of the composites were characterized by Fourier-transform infrared spectroscopy and water contact angle measurement. The antifungal activity of the composites was tested on both biofilm and planktonic cells with an XTT test 0 and 6 days after the composites’ preparation. The effect of the UDA+FAR combination on *C. albicans* filamentation was studied in agar containing 0.0125% and 0.4% UDA+FAR after 24 h and 48 h of incubation. The results showed the presence of UDA and FAR on the composite and decreases in the water contact angle and metabolic activity of both the biofilm and planktonic cells at both time points at non-toxic UDA+FAR concentrations. Thus, the modification of PMMA with a combination of UDA+FAR reduces *C. albicans* biofilm formation on dentures and could be a promising anti-*Candida* strategy.

## 1. Introduction

The essential problem of superficial fungal infections, which includes oral candidiasis, is of extremely high prevalence, in addition to the fact that 10% of patients develop a chronic form of the disease [1]. Chronic oral candidiasis occurs most often in people who wear dentures, and it is separated as a particular entity, *Candida*-associated denture stomatitis (CADS) [2,3]. One of the main causes of antifungal resistance and problems in the treatment of oral candidiasis is the ability of yeasts to form biofilms. A biofilm is defined as a community of microorganisms that have the ability to attach and multiply on some solid surfaces, such as dentures, which leads to the development of CADS [4]. Cells in this community, surrounded by an extracellular matrix, are protected from unfavorable conditions, as well as from the effects of antifungal drugs [5,6]. In addition to the biofilm matrix, other factors such as the overexpression of efflux pumps, changes in the ergosterol content and stress responses, and the presence of persister cells contribute to frequent failures in the management of biofilm-associated *Candida* infections [1,7]. Research is focused on different strategies to overcome the resistance of biofilm-forming microorganisms, including the nanotechnology approach, the identification of new compounds with anti-biofilm activity, alone or in combination, the turbimicin approach, the antifungal peptide produced by *E. faecalis*, and the combination of conventional antifungal drugs with any natural compounds or herbal extract [1,7]. Moreover, the formed biofilm constantly represents a reservoir and source of infection leading to chronicity, which is one of the biggest problems in the treatment of CADS. Additionally, dentures are usually made of poly(methyl methacrylate) (PMMA), which is a hydrophobic and rough material, and *Candida* yeasts adhere to such surfaces, multiply, and form biofilms [8]. *C. albicans* forms biofilms, which are the most abundant in mass, and this species is the most common causative agent of CADS [3,9]. In the form of biofilms, *C. albicans* is more resistant to conventional antifungal agents such as azole drugs and amphotericin B compared to planktonic cells [10].

Thus, it is crucial to develop new strategies for CADS treatment by preventing *Candida* biofilms from forming on dentures, which are a suitable substrate for microbial colonization. Hence, to overcome such undesirable properties of dentures, different approaches were proposed to make dentures antifungal, including surface modification as well as the incorporation of antifungal compounds into PMMA [11]. Surface modification implies coating the surface of the denture base resin with different materials such as 2-octyl cyanoacrylate [12], silane–silicon dioxide (SiO_2_) nanocomposite films [13], coatings containing zwitterion or hydrophilic monomers [11,14], and titanium dioxide (TiO_2_) [15,16,17]. Coating the denture base changes the surface properties from hydrophobic to hydrophilic, which further leads to decrease in *Candida* adhesion and biofilm formation. On the other hand, the coating may obliterate details of the inner denture surface and thus affect its retention in the oral cavity [11]. Therefore, to overcome the drawbacks of the coating, some researchers have investigated the incorporation of different antimicrobial compounds into the denture base. Thus, the incorporation of silver nanoparticles into PMMA has a successful antifungal effect without cytotoxicity, but discolorations have been reported as a drawback [18,19].

In addition, several natural products were reported to have stronger antifungal effects than conventional drugs and are less likely to induce microbial resistance [10,18]. Therefore, it is crucial to consider such natural products as novel drugs that will target biofilm-related infections [20].

Interestingly, research has shown that the incorporation of undecylenic acid (UDA), a natural compound, into PMMA provides strong antifungal properties to modified PMMA. However, UDA can be cytotoxic for human cells in concentrations of 6%, which will completely inhibit biofilm formation on dentures [21]. Current research on treating *Candida* infection aims to reduce the concentration of the antimicrobial drug by combining it with natural compounds. On the other hand, farnesol (FAR), also a natural compound, prevents *C. albicans* filamentation and biofilm formation and also has a synergistic interaction with some antifungal drugs [20,22,23]. Thus, the combination of FAR with other antifungal compounds could have an anti-*Candida* effect on resistant species at a relatively low total concentration of antimycotics. We therefore modified PMMA with a combination of UDA and FAR (UDA+FAR), aiming to obtain the antifungal PMMA_UDA+FAR composites in order to achieve a strong antifungal effect.

Furthermore, this study aims to develop new material by incorporating PMMA with a combination of UDA and FAR and to investigate the possibility of applying low concentrations of UDA in combination with FAR to obtain PMMA_UDA+FAR composites with a strong antifungal effect that will not be cytotoxic for human cells.

## 2. Materials and Methods

### 2.1. Sample Preparation

Cold polymerized acrylic resin, PMMA (Triplex Cold, Ivoclar Vivadent, Schaan, Liechtenstein), was incorporated with equal amounts of undecylenic acid (UDA) (ρ = 0.912 g/cm^3^) and farnesol (FAR) (ρ = 0.887 g/cm^3^) (1.5% UDA + 1.5% FAR *w*/*w*, 3% UDA + 3% FAR *w*/*w*, 4.5% UDA + 4.5% FAR *w*/*w*) to reach 3%, 6%, and 9% (*w*/*w*) of combination of those antifungal substances in total in PMMA (cured samples) according to the study by Petrović et al. [21,23]. Such PMMA_UDA+FAR composites were made as solid discs (Ø 20 mm, 2 mm in thickness) in Teflon molds for Fourier-transform infrared spectroscopy (FTIR) and contact angle measurements. For the XTT test (2,3-bis(2methoxy-4-nitro-5-sulfophenyl)-5-[(phenylamino)carbonyl]-2H-tetrazolium hydroxide test), the PMMA_UDA+FAR composites were prepared in the 24-well tissue culture plates (Falcon 353047) [21,23].

Prepared samples were stored in a hood at the room temperature for 24 h and were sterilized under a UV-C lamp for 15 min before the procedures. The PMMA_UDA+FAR composites made in the 24-well tissue culture plates were sterilized on the upper side while they were in the plates. For other experiments, samples were sterilized on both sides.

### 2.2. Physico-Chemical Characterization of PMMA_UDA+FAR Composites

#### 2.2.1. Fourier-Transform Infrared Spectroscopy (FTIR)

Fourier-transform infrared spectroscopy, FTIR (Spectrum One spectrometer (series: 69288, Perkin Elmer, Schwerzenbach, Switzerland), was employed for the chemical characterization of the PMMA_UDA+FAR surface. All spectra were registered in the range of 4000–400 cm^−1^, with 64 scans.

#### 2.2.2. Contact Angle Measurements

The physical characteristic of surface wettability of the prepared PMMA_UDA+FAR composite was established using the sessile drop method. The contact angle of 20 µL of double-distilled water (17_6 MW; 20_L) dropped on the PMMA_UDA+FAR surface was measured on a device (EasyDrop Standard, Krüss, Hamburg, Germany) equipped with a monochrome interline CCD camera. Data from a minimum of 3 measurements were presented as an average value followed by their standard deviation (SD).

### 2.3. Antifungal Characterization of Composites

#### 2.3.1. *Candida* Strain and Culture Conditions

For biofilm formation on PMMA, the *C. albicans* ATCC90028 reference strain, obtained from the American Type Culture Collection (ATCC, Manassas, VA, USA), was applied. Before the experiments, a strain from the stock (kept at −80 °C) was cultivated on Sabouraud 4% Glucose Agar (SGA; Sigma-Aldrich 84088, St. Louis, MO, USA).

#### 2.3.2. Biofilm Formation on PMMA_UDA+FAR Composites and the XTT Test

Based on previously recommended methods, we formed *C. albicans* biofilms on the PMMA_UDA+FAR composites [21,23,24].

Firstly, 400 µL of *C. albicans* suspension in RPMI 1640 medium, adjusted to 10^6^ cells/mL by counting it in the central square of a hemocytometer under a bright field microscope with a 40× objective [23], were dropped over the PMMA_UDA+FAR composites, which were prepared as solid discs on the bottom of the wells of the 24-well tissue culture plates. Samples were incubated for 24 h at 37 °C. The RPMI medium used in this case was RPMI 1640 with L-Glutamine without sodium bicarbonate (Sigma-Aldrich, R6504-10x1L).

After the incubation, 200 µL of the supernatant overlying the composites’ surface, which contained planktonic *C. albicans* cells, was carefully aspirated with a pipette and placed on a new 24-well plate. The rest of the suspension was taken out from the well, and the composite surface was washed with PBS to remove non-adherent cells and left to dry to remove any residual PBS. Further, per 200 µL of XTT/menadione solution (XTT (Cayman, Ann Arbor, MI, USA, CAS 111072-31-2) and menadione (Cayman, Ann Arbor, MI, USA, CAY15950-25g)) was added to the biofilm supernatant in the wells and composite surface in each of the wells to quantify metabolic activity of planktonic and biofilm (attached to the composite surface) *C. albicans* cells. With XTT/menadione solution, samples were incubated at 37 °C for 3 h. Further, the color intensity of the 100 µL XTT–menadione solution transferred into the new plate was measured with a microplate reader (TECAN Infinite M200, Tecan, Männedorf, Switzerland) at 490 nm. To quantify metabolic activity of both planktonic and biofilm *C. albicans* cells, the XTT test was performed in two different time intervals (immediately and 6 days after composite preparation; T0 and T6 time points).

### 2.4. Antifungal Susceptibility Test

#### 2.4.1. Determination of the Minimal Inhibitory Concentration (MIC)

To determine the minimal inhibitory concentration (MIC), the agar dilution method with modifications was used [23].

In a 50 mL polypropylene flat tube, we added different concentrations of UDA and FAR, which ranged from 0.0125, 0.025, 0.05, 0.1, 0.2, and 0.4% (*w/w*) in total (more precisely: 0.00625% UDA + 0.00625% FAR, 0.0125% UDA + 0.0125% FAR, 0.025% UDA + 0.025% FAR, 0.05% UDA + 0.05% FAR, 0.1% UDA + 0.1% FAR, and 0.2% UDA + 0.2% FAR, *w*/*w*) and mixed it with 40 mL of Yeast Peptone Dextrose (YPD) agar.

The agar prepared with defined UDA and FAR concentrations was transferred into the wells of the 24-well plates. Subsequently, 300 µL of *C. albicans* suspension with a concentration of 10^6^ *C. albicans* cells/mL were inoculated on the surface of each solidified agar (loaded with different UDA and FAR concentrations) and incubated at 37 °C for 24 h. After that, 100 µL of *C. albicans* suspension, which was overlying the agar surface, were placed into a new plate, and optical density was measured at a wavelength of 620 nm to determine the MIC value.

#### 2.4.2. Embedded Filamentation Test (EFT)

Alongside MIC detection, the effect of both substances on the *C. albicans* morphological changes was tested. In this test, we used agar embedded with the same minimum and maximum concentrations (0.0125 and 0.4% (*w/w*), respectively) of UDA and FAR as in the previously mentioned analysis. Briefly, a suspension made of 50 µL *C. albicans* cells with a concentration of 10^6^ *C. albicans* cells/mL and 5 mL of YPD agar that contained different concentrations of UDA and FAR was poured into a 50 mL Polystyrene Conical Tube (Sarstedt 352073); afterwards, this suspension was poured into Petri dishes (Ø 30 mm) and incubated at 37 °C for 24 h and 48 h [21,23].

The morphological characteristics and yeast and mycelial growth form of *C. albicans* cells in agar were investigated by optical microscopy (Nikon Eclipse Ti-E inverted microscope, Nikon Instruments Europe BV, Amsterdam, The Netherlands) after 24 h and 48 h.

### 2.5. Cytotoxicity Study

A cytotoxicity study of the PMMA_UDA+FAR samples was performed on the human epithelial cells, isolated from lung tissue (line A549, purchased from ATCC) in two time points: T0 (0 days after the PMMA_UDA+FAR composites’ preparation) and T6 (6 days after their preparation), according to the protocol from refs. [13,16]. Per composite, two PMMA_UDA+FAR discs were immersed in Falcon tubes containing 10 mL PBS and incubated for 24 h at 37 °C, followed by shaking for 10 s on a vortex in order to obtain conditioned media. Cells were cultured in the RPMI-1640 medium supplemented with 10% fetal bovine serum and 2% 5000 U mL^−1^ penicillin, 5 mg mL^−1^ streptomycin, and 10 mg mL^−1^ neomycin (Sigma-Aldrich) at 37 °C for 24 h. The cell density was adjusted to 4000 cells per well in a 96-well plate. A total of 100 µL of media containing 50% of the fresh growth media and 50% (*v*/*v*) of the “disc conditioned media” were added to the cells and incubated for an additional 24 h. Then, 100 µL of 3-(4,5-dimethylthiazol-2-yl)-5-(3-carboxymethoxypenyl)-2-(4-sulfophenyl)-2H-tetrazolium (MTS) solution (diluted 6 times in the medium) was added to every well and incubated for 2 h at 37 °C. The absorbance of the formazan product was measured with a microplate reader (Tecan Infinite M200, Tecan, Männedorf, Switzerland) at a wavelength of 490 nm. The MTS test were performed in four repetitions [21,23]. 

### 2.6. Statistic

The results obtained from the contact angle, XTT assay, and cytotoxicity tests were given as means ± SDs. ANOVA, followed by a post hoc Tukey’s test, was applied to study the significant differences between more than two defined groups and analyze associations between continuous variables with Pearson’s correlation coefficient (r).

## 3. Results and Discussion

### 3.1. Physico-Chemical Characterization of PMMA_UDA+FAR Composites

#### 3.1.1. FTIR (Chemical Characterization of PMMA_UDA+FAR Composite Surfaces)

**Figure 1:**
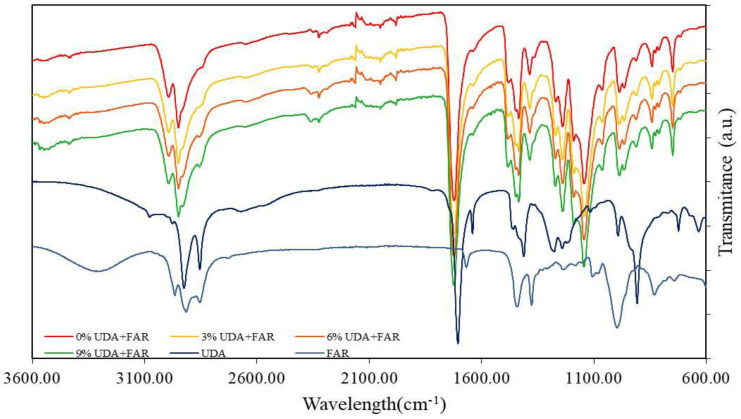
Fourier-transform infrared spectra of undecylenic acid (UDA), farnesol (FAR), poly(methyl methacrylate) (0% UDA+FAR), and composites with 3%, 6%, and 9% UDA+FAR.

In order to determine the presence of UDA and FAR on the surfaces of the PMMA_UDA+FAR composites, FTIR measurements were performed. All characteristic peaks for PMMA (Figure 1) and peaks for both antimicrobial compounds (UDA and FAR) had been identified and compared with the spectra of the PMMA_UDA+FAR composites with different UDA+FAR concentrations. In the PMMA spectrum, we identified bands at 2985 cm^−1^ and 2964 cm^−1^, assigned to the CH stretching vibration from the -CH2 and -CH3 groups, respectively, as well as a characteristic vibration band of PMMA visible as a sharp, intense peak at 1727 cm^−1^, which corresponds to the stretching vibrations to the ester group (C=O) [21,23,24]. Characteristic peaks of UDA, such as the C=C (at 1642 cm^−1^ and 908 cm^−1^) [21], =C–H (at 3078 cm^−1^) [21], and O–H (at 1411 cm^−1^) [21] groups, were observed in the UDA spectrum. The C=C peaks of UDA were confirmed in the spectrum of all PMMA_UDA+FAR composites with all the tested concentrations of antimicrobial compounds, indicating the presence of UDA on the composite’s surface. The =C–H bend of UDA was not clearly observed in the spectrum of the PMMA_UDA+FAR composites. Our previous study [21] reported that the =C–H bend of UDA was identified in the PMMA–UDA composites with 9% and 12% of UDA only as a weak peak shifted towards lower wavelengths (3074 cm^−1^), while it was not seen in the composites with lower concentration of UDA into PMMA (3% and 6%) [21]. Since, in our study, the highest concentration of UDA into the PMMA_UDA+FAR composites was 4.5% (half of 9% of total UDA and FAR), the =C–H bend of UDA was not pronounced, and this is in agreement with the study with 3% and 6% of UDA into PMMA [21]. In the spectrum of pure FAR, characteristic peaks were identified, such as stretching vibrations of the O-H group at 3344 cm^−1^, and bands assigned to asymmetric stretching vibrations of the -CH3 group at 2969 cm^−1^ and -CH2 groups at 2919 cm^−1^ [23,25], while the C-H bending mode was observed at 1377 cm^−1^ [26]. Our results (Figure 1) showed that characteristic peaks of UDA and FAR, which are not characteristic of PMMA, were observed in the spectrum of the PMMA_UDA+FAR composites, confirming the presence of both antimicrobial substances, UDA and FAR, on the PMMA surface. The intensity of the characteristic peaks of both UDA and FAR in the PMMA_UDA+FAR composites corresponded to the UDA and FAR amounts in composites. Namely, with an increase in the concentration of the antifungal substance in the acrylate, the intensity of the peaks also increases. Furthermore, the absence of new peaks in the PMMA_UDA+FAR composites in relation to PMMA, FAR, and UDA was observed. This fact indicates that the interactions between the polymer matrix and the antimicrobial substances are physical, achieved through hydrogen bonds, without new chemical bonding between functional groups of UDA, FAR, and PMMA [24].

#### 3.1.2. Contact Angle Measurement (Physical Characterization of PMMA_UDA+FAR Composite Surfaces)

**Figure 2:**
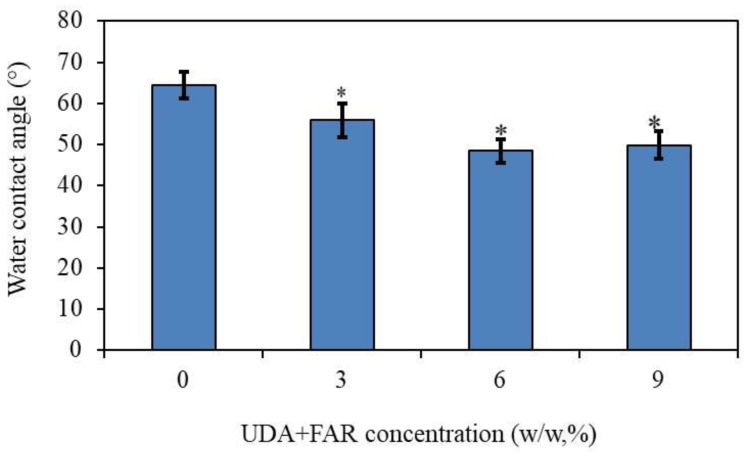
Water contact angle on the surface of the composites with 0%, 3%, 6%, and 9% of the total concentration of both antimicrobial compounds undecylenic acid (UDA) and farnesol (FAR). Asterisks above the columns show significant differences among the groups and compared to the controls (*p* < 0.05, Tukey’s test). The results are presented as means ± SDs.

The measurement of the water contact angle on the PMMA_UDA+FAR composite’s surface was performed to study whether the incorporation of UDA+FAR into the polymer matrix changes the surface’s wettability. The results showed (Figure 2) that the water contact angle significantly decreased in the PMMA_UDA+FAR composites in all tested concentrations of the antimicrobial compounds to 56.8° for composites with 3% UDA+FAR compared to the composites with 0% UDA+FAR (64.38°) (*p* < 0.05). The incorporation of UDA+FAR into PMMA changed the surface properties to become more hydrophilic, even though these antimicrobial compounds are hydrophobic. The same finding has been reported in the PMMA composites incorporated only with UDA [21] as well as only with FAR [23]. This finding can be explained by the orientation of UDA and FAR molecules on the PMMA surface. Specifically, UDA molecules are composed of a polar head and nonpolar tail positioned in such a way that a polar head of UDA orients towards the surface, while the tail would be orientated out of the surface [21,23]. Surface modification into a more hydrophilic one, as compared to pure PMMA, presents an important step because it can inhibit the fungal colonization of polymeric biomaterials, which is the first critical step of biofilm formation [8,24].

### 3.2. Antifungal Characterization of Composites

#### 3.2.1. Biofilm Formation and XTT Assay on Biofilm Cells

**Figure 3:**
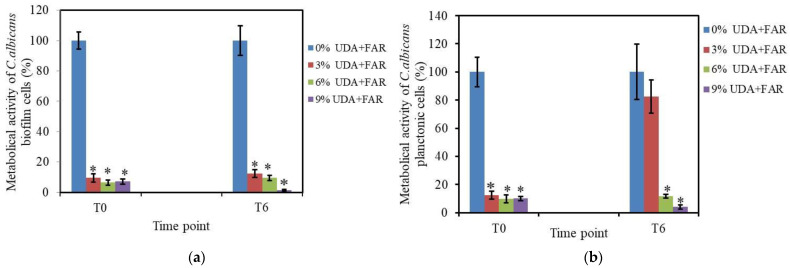
Comparison of a metabolically active *Candida* (*C.*) *albicans* biofilm (**a**) and planktonic (**b**) cells after incubation with poly(methyl methacrylate) (PMMA) modified with the combination of equal concentrations of tundecylenic acid (UDA) and farnesol (FAR) (0%, 3%, 6%, and 9% UDA+FAR in total) in two points: 0 days (T0) and 6 days (T6) after PMMA_UDA+FAR composite preparation. The asterisks denote significant differences (*p* < 0.05) compared to the PMMA control.

Furthermore, to test the antifungal surface properties of PMMA modified with 3%, 6%, and 9% UDA+FAR, we measured the metabolic activity of *C. albicans* cells attached to the composite surface (biofilm cells) after 24 h of incubation with the PMMA_UDA+FAR composites. Quantification of metabolic activity of those biofilm cells was performed by the XTT test in two time points (0 days and 6 days after the composite preparation, denoted T0 and T6, respectively) in order to study whether antifungal surface properties change with time. Even with the lowest concentration, 3% UDA+FAR, the percentage of metabolically active biofilm cells on the PMMA_UDA+FAR composites significantly decreased to ~9.5% and ~12.4% in the T0 and T6 intervals, respectively, compared to 100% metabolically active biofilm cells on PMMA with 0% UDA+FAR (*p* < 0.001) (Figure 3a). In T6, biofilm formation was decreased on all the PMMA_UDA+FAR composites with less than 12% of the metabolically active biofilm cells, indicating that composites maintain strong anti-*Candida* activity of the surface with time. Furthermore, our results showed that the concentration of UDA+FAR required to reduce more than 90% of metabolically active biofilm cells was lower in the combination of these substances (3% UDA+FAR in total in PMMA, meaning per 1.5% UDA and 1.5% FAR) than when these compounds were employed individually. More precisely, a previous study showed that the incorporation of 3% FAR into PMMA did not affect biofilm formation nor planktonic *C. albicans* cells [23], while 3% UDA into PMMA had a weak antibiofilm effect and no effect on planktonic cells [21]. Specifically, it has been reported that individually 6% UDA in PMMA [21] and 6% FAR in PMMA [23] were needed to reduce 90% metabolically active biofilm cells. Alongside the fact that the incorporation of 6% UDA into PMMA was very effective to inhibit biofilm formation, it had been reported that the same concentration affected human cell viability (i.e., ~53% of cells remained viable 6 days after composite preparation) [21]. Therefore, it was of interest to use the UDA concentration in PMMA that will have a strong antibiofilm effect with a non-toxic concentration. A combination of two or more antimicrobial agents at their non-toxic concentrations in order to maintain or increase the overall antifungal effectiveness has been a common practice [27]. It has been demonstrated that FAR in combination with conventional antifungal agents increases its effectiveness [22]. To date, there has been no report about the interaction of FAR with natural compounds such as UDA. Thus, we hypothesized that non-toxic concentrations of UDA in combination with FAR in PMMA could still achieve anti-*Candida* effects. Moreover, our results confirmed that in the combination of UDA+FAR, antibiofilm effects have been achieved with a lower, non-cytotoxic concentration of both substances (1.5% UDA and 1.5% FAR) compared to the effects of UDA or FAR individually [21,23]. Importantly, numerous studies have confirmed that the increase in hydrophilicity of the surface of the denture base resin strongly correlates with the reduced microbial adhesion [13,23,28,29,30]. Our results showed a positive correlation (r = 0.83) between the contact angle values and metabolic activity of *C. albicans* biofilm cells on the PMMA_UDA+FAR composites. In fact, alongside surface wettability, other factors, such as the presence of UDA and FAR, namely their chemical groups at the composite’s surface affected the *C. albicans* adhesion on the surface. Klotz et al. reported higher *C. albicans* adherence to the hydrophobic surfaces (determined by the contact angle measurement) and a nearly linear relationship between the number of *C. albicans* adhering per unit area and the hydrophobicity of polymers [31,32]. Numerous studies have shown that changing the surface topology into a hydrophilic one (such as hydrophilic surface modification by the silica coating, TiO_2_ thin films, crosslinkable co-polymer containing sulfobetaine methacrylamide (SBMAm), and atmospheric pressure cold plasma-induced surface modification), decreases adhesion and biofilm formation to the surface [31,33,34,35]. Puri et al. reported that phosphate incorporation into an acrylic resin denture material by monomer substitution improved hydrophilicity, but dimensional and color stability were disrupted [36]. Therefore, the development of antifungal material with a fungicidal effect while preserving dimensional and color stability could be of interest.

#### 3.2.2. XTT Assay on Planktonic Cells

The planktonic *C. albicans* cells are not attached to the surface of the composites, but free-floating in a medium. In order to test if planktonic cells of *C. albicans* are affected by the UDA+FAR released from the PMMA_UDA+FAR composites, we measured the metabolic activity of the planktonic cells by the XTT test. The percentage of metabolically active planktonic cells was <12.5% in all the tested concentrations of UDA+FAR in the T0 point, while in the T6 time point, antifungal properties of the PMMA_UDA+FAR composites was achieved with the concentration of 6% UDA+FAR in PMMA (Figure 3b). On the other hand, when those substances were incorporated into PMMA individually, the antifungal effect on the planktonic cells was not as strong as our results. Specifically, it has been reported that for individually incorporated 3% UDA in PMMA [21], the percentage of metabolically active planktonic cells was <60%, while in the case with 3% FAR in PMMA, there was no effect on the metabolic activity of planktonic cells at the T0 time point [23]. Thus, the combination of UDA+FAR into PMMA showed stronger anti-*Candida* activity on the planktonic cells than when being incorporated alone [21,23].

Moreover, the fact that there were fewer metabolically active planktonic and biofilm cells after the incubation with the composites modified with the combination of UDA+FAR than separately with UDA and FAR suggests synergistic activity of those substances. UDA has a fungicidal effect on *Candida* and inhibits biofilm formation by inhibiting morphogenetic transition of *C. albicans* from yeast to hyphal form, which is the initial step of biofilm formation [21,37,38]. Although the mode of action of FAR is not yet clear, it seems to be a complex one, including several mechanisms such as growth-inhibitory and apoptosis-promoting effects [20]. Furthermore, FAR in combination with antifungal agents enhances its efficacy [20]. Thus, Katragkou et al. reported that FAR exerts a synergistic or additive interaction with micafungin, fluconazole, and amphotericin B against *C. albicans* biofilm, most probably by substantial accumulation of reactive oxygen species (ROS), which further causes apoptotic cell death [20]. Furthermore, FAR also prevents filamentation and, hence, consequent biofilm formation [39,40].

*C. albicans* biofilm formation occurs in three stages: early, intermediate, and mature [27]. The initial step of biofilm formation is the adhesion of single cells to the surface due to electrostatic and hydrophobic interactions among cells and surfaces. This early stage of biofilm formation, occurring in the first 24 h, is followed by proliferation and early-stage filamentation of the adhered cells towards the formation of mature biofilms (48 h after the adhesion) [6]. A mature biofilm consists of several layers of polymorphic cells, including hyphal, pseudohyphal, and round yeast cells, encased in an extracellular matrix, which protects cells from chemical and physical injury, making them more resistant to antimicrobial compounds. The last step of biofilm development is the dispersion of round yeast cells from the biofilm, which further seeds new sites [6]. Therefore, inhibition of early-stage biofilm is crucial to prevent the development of mature *C. albicans* biofilm, which is more difficult to treat because the ECM protects cells from chemical and physical injury and lowers the diffusion of antimicrobial drugs, making them more resistant to the antimicrobial compound. Thus, in our study, the inhibition of *C. albicans* biofilm formation, detected by measuring the metabolic activity of biofilm cells, could be related either to interference with the adhesion of growing cells to the hydrophilic surface of the PMMA_UDA+FAR composites or to the inhibition of morphogenetic transition of yeast cells to hyphae, or both.

### 3.3. Antifungal Characterization of UDA+FAR

#### 3.3.1. Antifungal Susceptibility Test—Determining Minimal Inhibitory Concentration of UDA+FAR

**Figure 4:**
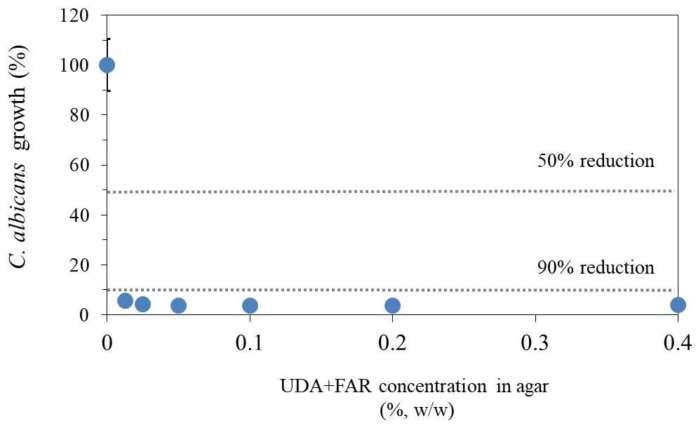
The effect of the combination of undecylenic acid (UDA) and farnesol (FAR) on *C. albicans* growth after 24 h of incubation on the agar surface loaded with different UDA+FAR concentrations. The results are given as a mean ± standard deviation of optical density reading at a wavelength of 620 nm.

For better understanding the mode of action of the UDA+FAR combination, we employed two different assays to study the effect of “pure” UDA+FAR without PMMA on *C. albicans*’ growth. Firstly, the UDA+FAR combination was dispersed in an agar, and already at the lowest concentrations of UDA+FAR, 0.0125% in total (more precisely: 0.00625% UDA + 0.00625% FAR), the reduction of more than 90% *C. albicans*’ growth was obtained (Figure 4). This is very different from the results when those substances were tested separately [21,23]. Namely, it has been reported that UDA [21] or FAR [23] individually dispersed in an agar reduced 90% of *C. albicans*’ growth at the concentration of 0.0125%.

#### 3.3.2. Embedded Filamentation Test

**Figure 5:**
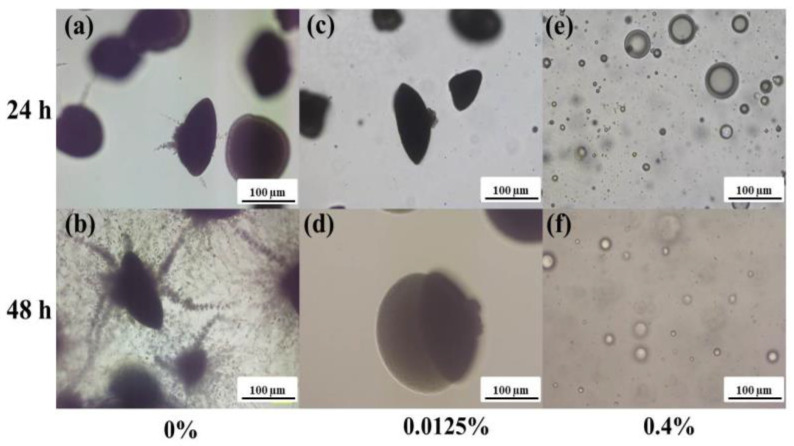
Representative optical micrographs showing morphologic changes of *C. albicans* cells embedded in the agar without the combination of undecylenic acid (UDA) + farnesol (FAR) (control, 0% UDA+FAR, (**a**,**b**)), with 0.0125% UDA+FAR (**c**,**d**) and 0.4% UDA+FAR (**e**,**f**) after 24 h (**a**,**c**,**e**) and 48 h (**b**,**d**,**f**) of incubation. The arrows show spindle-shaped colonies with sporadic hyphal/pseudohyphal forms on the periphery and lateral yeasts in agar loaded with 0.0125% UDA+FAR in total (**c**,**d**). The images were taken with a magnification of 40×.

Moreover, the embedded filamentation tests showed that at the lowest concentration of the UDA+FAR (0.0125%), *C. albicans* grew as spindle-shaped colonies (without filamentous forms) after 24 h of incubation (Figure 5). Filamentation occurred after 48 h of incubation at 0.0125% UDA+FAR. However, at the highest tested concentration, 0.4% UDA+FAR (more precisely 0.2% UDA + 0.2% FAR), *C. albicans* growth was inhibited at both time points (24 h and 48 h after the incubation), and only spherical forms were detected. After the incubation of *C. albicans* with 0.4% of only UDA in the agar, the same formation of spherical forms had been reported, probably due to the deterioration of intracellular material after disintegration of the yeasts’ cells [21]. Actually, the study of only UDA in the same test reported the inhibition of switching *C. albicans* cells to hyphal form at 0.0125% UDA, while 0.4% UDA exhibited fungicidal effects [21]. In our study, we reached the same effect when UDA was combined with FAR at both tested concentrations after both 24 h and 48 h of incubation. On the other hand, when *C. albicans* was exposed only to FAR at both concentrations of 0.0125% and 0.4%, after 24 h of incubation, the spindle-shaped colonies (without filamentous forms) were observed, while filamentation occurred after 48 h of the incubation at both tested concentrations [23]. Although FAR also blocked filamentation, FAR cannot inhibit the elongation of already existing hyphae. Therefore, cells are sensitive to FAR only for a limited time, and, consequently, filamentation occurred after 48 h of *C. albicans* exposure only to FAR [23,41]. Thus, the results of the filamentation test in this study confirmed that the combination of both UDA and FAR with lower concentrations (per 0.0625% and 0.2%) had the same effect as when UDA was individually employed at 100% higher concentrations (0.0125% and 0.4%, respectively) [21] after both 24 h and 48 h of incubation. This can be explained by the additive effect of both substances, such as the fungicidal effect of UDA and filamentation inhibition of both UDA and FAR.

In the agar dilution method, according to previous studies [21,23], we used UDA and FAR concentrations of 0.0125, 0.025, 0.05, 0.1, 0.2, and 0.4% in total [21]. Based on this test, we determined the MIC (for planktonic *C. albicans* cells). Further, for the embedded filamentation test, we chose the minimum and maximum concentrations from the agar dilution method (0.0125% and 0.4%). Based on the fact that the concentration of antifungal agents needed to treat biofilm cells is usually much higher than for planktonic populations (normally up to 100–1000 times higher) [42], we chose to test different UDA+FAR concentrations in PMMA based on the MIC. Moreover, we took the concentration of 3% UDA+FAR as a starting point in accordance with the previous studies of UDA [21] and FAR [23] into PMMA.

### 3.4. Toxicity of PMMA_UDA+FAR Composites

**Figure 6:**
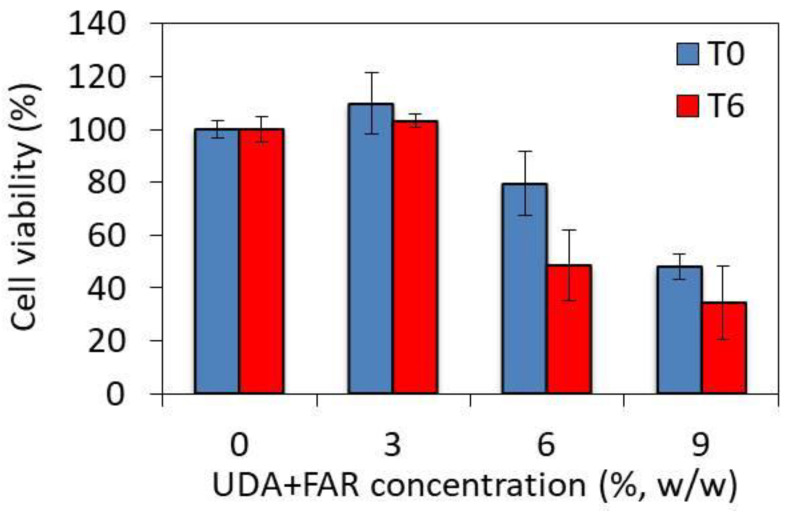
The viability of human A549 cells incubated with media after the immersion of PMMA with the combination of undecylenic acid (UDA) and farnesol (FAR) in different concentrations, (in total, 3%, 6%, and 9% *w*/*w* UDA+FAR, more precisely per 1.5%, 3%, and 4.5% *w*/*w* of UDA and FAR, respectively), measured by the MTS test in two studied time points, T0—0 days—and T6—6 days after the PMMA_UDA+FAR disc preparation. The PMMA_UDA+FAR combinations were immersed for 24 h and six days longer for the T0 and T6 time points.

Both UDA and FAR are used as antifungal agents to prevent biofilm formation. In order to be used for medical purposes, low toxicity of the UDA+FAR composite to human cells must be demonstrated. We therefore performed a preliminary toxicity evaluation and studied the viability of human A549 cells seeded on the composites incorporating different UDA+FAR concentrations using the MTS viability assay at two time points. At T0, i.e., right after the preparation of the composite, the cell viabilities measured for the UDA+FAR concentration of 3% and for the composite without UDA+FAR were the same (Figure 6). At a concentration of 9% of UDA+FAR, a drop in cell viability of 52% was observed. At T6, 6 days after the preparation of the composites, no decrease in cell viability was observed for 3% of UDA+FAR, while the cell viability significantly dropped for the UDA+FAR concentrations above 3%. Based on these preliminary toxicity results, the composite loaded with the UDA+FAR concentrations of up to 3% is promising for medical applications.

## 4. Conclusions

This study reported, for the first time, that FAR in combination with UDA incorporated into the cold polymerized acrylic resin (PMMA) may allow for obtaining antifungal surface properties. UDA and FAR released from the PMMA_UDA+FAR composites affected the metabolic activity of planktonic cells. Both UDA and FAR are presented on the composite surfaces, enabling the hydrophilic surface of developed PMMA_UDA+FAR composites and changing the surface properties as compared to native PMMA. Inhibition of the early stage of biofilm formation was achieved with the PMMA_UDA+FAR composites having non-toxic concentrations of UDA+FAR of 3%. This inhibition was observed at both time points. In summary, we can point out that the developed composites of PMMA with the combination of UDA and FAR have strong antifungal effects at non-toxic concentrations, which could form a good basis for solving the issue of CADS.

## Data Availability

The original contributions presented in the study are included in the article, further inquiries can be directed to the corresponding author.
